# Cancer and the Web

**DOI:** 10.1002/cfg.65

**Published:** 2001-02

**Authors:** Mark Robert Albertella

**Affiliations:** KuDOS Pharmaceuticals Ltd 327 Cambridge Science Park Milton Road Cambridge CB4 0WG UK

## Abstract

The applications of functional genomics, proteomics and informatics to cancer research have yielded a tremendous amount of information, which is growing all the time. Much of this information is available publicly on the Internet and ranges from general information about different cancers from a patient or clinical viewpoint, through to databases suitable for cancer researchers of all backgrounds, to very specific sites dedicated to individual genes or molecules. A simple search for ‘cancer’ from a typical Web browser search engine yields more than half a million hits; an even more specific search for ‘leukaemia’ (>40 000 hits) or ‘p53’ (>5700 hits) yields far too many hits to allow one to identify particular sites of interest. This review aims to provide a brief guide to some of the resources and databases that can be used as springboards to home in rapidly on information relevant to many fields of cancer research. As such, this article will not focus on a single website but hopes to illustrate some of the ways that postgenomic biology is revolutionizing cancer research. It will cover genomics and proteomics approaches that have been applied to studying global expression patterns in cancers, in addition to providing links ranging from general information about cancer to specific cancer gene mutation databases.


**Comparative and Functional Genomics**
*Comp Funct Genom* 2001; **2**: 35–43.

**Website Review**

### Cancer and the web

Mark Robert Albertella*
*KuDOS Pharmaceuticals Ltd, 327 Cambridge Science Park, Milton Road, Cambridge CB4 0WG, UK*

*Correspondence to:
M. R. Albertella, KuDOS Pharmaceuticals Ltd, 327 Cambridge Science Park, Milton Road, Cambridge CB4 0WG, UK.
E-mail: malbertella@kudospharma.co.uk*

### Abstract

**The applications of functional genomics, proteomics and informatics to cancer research have yielded a tremendous amount of information, which is growing all the time. Much of this information is available publicly on the Internet and ranges from general information about different cancers from a patient or clinical viewpoint, through to databases suitable for cancer researchers of all backgrounds, to very specific sites dedicated to individual genes or molecules. A simple search for 'cancer' from a typical Web browser search engine yields more than half a million hits; an even more specific search for 'leukaemia' (>40 000 hits) or 'p53' (>5700 hits) yields far too many hits to allow one to identify particular sites of interest. This review aims to provide a brief guide to some of the resources and databases that can be used as springboards to home in rapidly on information relevant to many fields of cancer research. As such, this article will not focus on a single website but hopes to illustrate some of the ways that postgenomic biology is revolutionizing cancer research. It will cover genomics and proteomics approaches that have been applied to studying global expression patterns in cancers, in addition to providing links ranging from general information about cancer to specific cancer gene mutation databases. Copyright © 2001 John Wiley & Sons, Ltd.**

### A starting point for new cancer researchers

There is a good general introduction to cancer biology at the National Centre for Biotechnology Information (NCBI) *Genes and Disease* site (**http://www.ncbi.nlm.nih.gov/disease/Cancer.html**). This site has links to several common cancers and cancer genes which contain concise summaries of the disease, its impact and what is known about the underlying genetics. Each description is exemplified by one or two genes known to be important in that disease, e.g. the colon cancer entry describes the roles of the mismatch repair genes MSH2, MSH6 and MLH1. Links to OMIM, LocusLink and EntrezGenome databases (see below) are included to access further information about these genes.

*OncoLink* (**http://oncolink.upenn.edu**) from the University of Pennsylvania Cancer Centre is an excellent source of news and literature information about cancer. The 'Disease menus' link leads to specific sets of information about each cancer, generally from a more clinical viewpoint. The whole site can also be searched for individual or combined terms, or just the Cancer News section, which again focuses on clinical studies more than basic research. There are also links to FAQs, clinical trial information, information for patients, book reviews and more.

### Cancer genes and genetics

There are many ways to focus in rapidly on general and cancer-related information about an individual gene using the resources at the NCBI—an impressively curated, comprehensively integrated set of resources that allow a rapid and thorough trawl of gene-related data. All of these resources can be queried using a gene name (e.g. $\beta$-catenin), gene symbol (CTNNB1), accession number, or more general query term (e.g. catenin). All resources are

Copyright © 2001 John Wiley & Sons, Ltd.

linked between OMIM, LocusLink and UniGene where appropriate. The oncogene $\beta$-catenin has been used as an example query term for the following summaries.

### Online Mendelian inheritance in man (OMIM)

**http://www.ncbi.nlm.nih.gov/entrez/query.fcgi?db = OMIM**

The OMIM resource is a catalogue of genes and genetic disorders and contains locus information with a detailed review of the literature relevant to the queried gene and its genetic characterization (e.g. $\beta$-catenin, entry 116806). These reviews can be quite comprehensive and are kept well up to date. References are linked to PubMed and internally linked to other OMIM entries where appropriate. Selected allelic variations, in particular disease associated mutations culled from the literature, are detailed with links to the relevant literature.

Genes and loci in the vicinity of the queried term (e.g. 3p22–p21.3) can be viewed in the *OMIM Gene Map* tables, organized by chromosomal location and listing gene name and symbol and links to the map viewer. Any disorders associated with the entries are indicated (e.g. for $\beta$-catenin: colorectal cancer; hepatoblastoma; pilomatricoma; ovarian carcinoma, endometrioid type) and the gene mapping methods used to define that locus are supplied. The *OMIM Morbid Map* can also be queried by disease term to flag all genes curated as being involved in that disease.

### UniGene

**http://www.ncbi.nlm.nih.gov/UniGene/**

UniGene attempts to cluster EST and cDNA sequences corresponding to a gene in an automated manner. For a given search term, UniGene returns a comprehensive entry for the cluster (e.g. $\beta$-catenin Hs. 171271) listing all constituent ESTs, with brief descriptions of library source and sequence read (e.g. 3′ sequence, including poly A tail, etc.). The UniGene entry also links to gene and complete mRNA sequences and includes a selected list of homologues, ranked by identity at the protein level (with links to database entries). It also supplies cytogenetic information (with a link to a close-up of the chromosomal region via OMIM) and expression information, defined by the cDNA library source of each of the clustered ESTs, in addition to links to SAGE data (see below). One drawback of this automated system that should be noted is that it can lead to some false clustering, due to partial or incomplete cDNA sequence data.

### LocusLink

**http://www.ncbi.nlm.nih.gov/LocusLink/**

LocusLink provides a curated information resource centred around the queried genetic locus (e.g. $\beta$-catenin entry, 1499). It collates a tremendous amount of useful information, such as literature aliases, phenotype descriptions, chromosomal mapping information and context and STS markers in the genomic region. Protein sequence domains are listed (such as armadillo/$\beta$-catenin-like repeats) and are linked to the CD-Browser entry (e.g. ARM). This entry lists all proteins with this domain that can be individually or collectively selected and aligned from the site. Alternatively, complete protein or nucleotide sequence links can be BLASTed directly from the site.

### GeneCard

**http://bioinformatics.weizmann.ac.il/cards/**

GeneCard is a database of human genes and products collating information from a variety of different resources, provided by the Weizmann Institute, Israel. GeneCard links back to nucleotide and protein sequences, UniGene, OMIM, mammalian homologues and cytogenetic location.

### Tumour Gene Database (TGD)

**http://condor.bcm.tmc.edu/oncogene.html/**

The Tumour Gene Database aims to provide collections of facts (culled from literature reports) about gene entries. This site can be searched by gene name, or by fact term—e.g. if you are looking for facts involving cisplatin. The database is not very comprehensive, but there is a wealth of information on some genes (e.g. BRCA1), and if your gene of interest is present, then TGD is an excellent resource for literature searching with concise descriptions of the relevant fact from a particular paper. Facts are returned grouped under a variety of headings, e.g. cell cycle, clinical, function, phenotype, reviews, tumour incidence, and are linked to the PubMed entry of the reference paper.

### Individual gene mutation databases

There are many databases that catalogue mutations identified in common oncogenes or tumour

Copyright © 2001 John Wiley & Sons, Ltd.

suppressors (e.g. the MSH2 database: **http://www.nfdht.nl/database/msh2.htm**). LocusLink and GeneCard entries (see above) commonly include links to individual gene mutation databases, where available. There is also a list of many gene mutation databases at the Human Gene Mutation Database (**http://archive.uwcm.ac.uk/uwcm/mg/docs/oth_mut.html**).

### Cancer genomics

### The Cancer Genome Anatomy Project (CGAP)

The stated aim of CGAP (**www.ncbi.nlm.nih.gov/ncicgap**) at the National Cancer Institute is to define the molecular anatomy of a cancer cell, in particular to define which genes are expressed in cancers. This information has been obtained from the sequencing of cDNA libraries derived from different cancer and normal samples. The site is divided into five sections: the human Tumor Gene Index; the mouse Tumor Gene Index; the Molecular Profiling Initiative; the Genetic Annotation Initiative; and the Cancer Chromosome Aberration Project (Figure 1).

The human and mouse *Tumour Gene Indexes* include the cDNA sequence databases generated from an impressive range and number of tumour and normal tissue and cell libraries. The resource is intended to identify which genes are expressed in different tumours and to allow the identification of novel genes. The cDNA libraries used can be searched or browsed and are well documented for numbers of clones sequenced per library, source and preparation method of library, normalization method (if used) and purchasing details. The cDNA libraries can be searched using the GeneExpress tool for a particular gene by UniGene cluster ID (see above). All cDNA libraries found to contain this sequence are returned, with the abundance of the sequence indicated (e.g. three copies in 15 000 sequences), although the libraries are not ranked in any discernible order, such as tissue type or sequence abundance. The site has two tools for comparing sequence expression profiles between libraries: Digital Differential Display and cDNA Xprofiler. However, the cDNA library sequencing approach for comparing gene expression patterns is quite limited, for two main reasons. First, many of the libraries used have been normalized, and thus have distorted transcript abundance levels. Second, the depth of sequencing of each library is generally limited, leading to skewed relative abundances of constituent sequences. This renders much of the comparative expression analysis of limited value.

The *Molecular Profiling Initiative* aims to provide a resource for collating the technical requirements for analysis of cancer and tissue specimens; however, there is currently no data in this branch of the site. The *Genetic Annotation Initiative* (GAI) aims to identify variation (SNPs) in genes important in cancer. The GAI utilizes data-mining tools to identify candidate variation from public sequence databases, which are then experimentally analysed to determine whether this variation is genuine or a sequencing artefact. This approach is currently in early stages and is inherently limited by the input EST data available and depth of coverage problem. The GAI will undoubtedly be overshadowed by the much more ambitious and thorough approaches proposed by Professor Mike Stratton, joint head of the *Cancer Genome Project*. This project (see **http://www.sanger.ac.uk/Info/Press/991020.shtml** for press release), established by the Wellcome Trust and to be sited at the Sanger Centre, aims to build on the Human Genome Project data to identify every gene mutated in cancer, and will without doubt become a central resource for the cancer community in the future.

The *Cancer Chromosome Aberration Project* has compiled a database of the chromosomal aberrations found in different cancers. This database can be searched by the *Recurrent Chromosome Aberrations* link and this search can be optionally restricted by chromosome, arm, band, tissue type and/or neoplasm. For example, a search for 'Wilms' tumour' reveals a table of the recurrent abnormalities observed in Wilms' tumour patients, indicating band, abnormality, neoplasm and number of cases in the database. In this example (Figure 2) many cases involve deletions of the 11p11–14 region. These links can be followed to the locus map of this region in OMIM, where it can be seen that the Wilms' tumour susceptibility genes WT1 and WT2 reside.

### Mouse Tumor Biology Database (MTB)

**http://tumor.informatics.jax.org/FMPro?-db = TumorInstance&-format = mtdp.html&-view**

This Jackson Laboratory site was reviewed in Issue 2 of this journal (Wixon, 2000), so I will just mention it briefly here. MTB (Bult *et al.*, 2000) is searchable by tumour type, tissue/organ, literature reference, gene or genetic alteration to link to an

Copyright © 2001 John Wiley & Sons, Ltd.

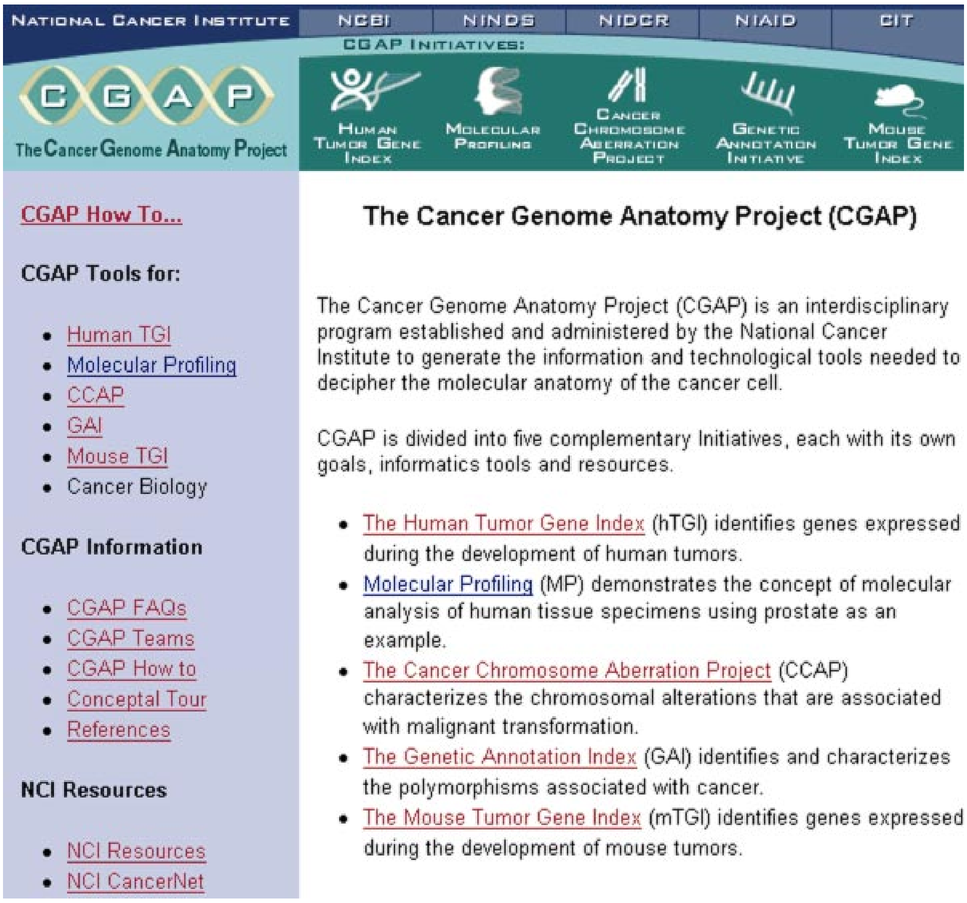

**Figure 1.** The Cancer Genome Anatomy Project home page. The links to the five constituent Initiatives can be followed from the top bar, the side bar, or the main panel. The *CGAP How To…* link at the top of the side bar gives useful pointers for getting the most out of the CGAP site. Reproduced with the kind permission of the National Library of Medicine **http://www.ncbi.nlm.nih.gov**

impressive array of information available on mouse tumours. This is an excellent resource to investigate mouse models and homologues of your gene of interest.

### Transcriptomics

There are resources on the Internet for at least three approaches to gene expression profiling (transcriptomics) in tumours: cDNA library sequencing; Serial Analysis of Gene Expression (SAGE); and microarray analysis.

The approach of mass sequencing of cDNA libraries has been extensively used by CGAP, as described above. The principal limitation of this approach is that the depth of coverage required to get statistical sampling of expression levels is very high, and that many cDNA library preparations are generated by normalization, which, by definition, alters the relative abundance of constituent sequences. These limitations can be partially resolved by use of the SAGE technique (see below), which can readily generate the greater depths of coverage required for meaningful differential analysis.

### Serial Analysis of Gene Expression (SAGE)

The SAGE technique, developed by Bert Vogelstein, Kenneth Kinzler and colleagues at Johns Hopkins University (Velculescu *et al.*, 1995), generates

Copyright © 2001 John Wiley & Sons, Ltd. 

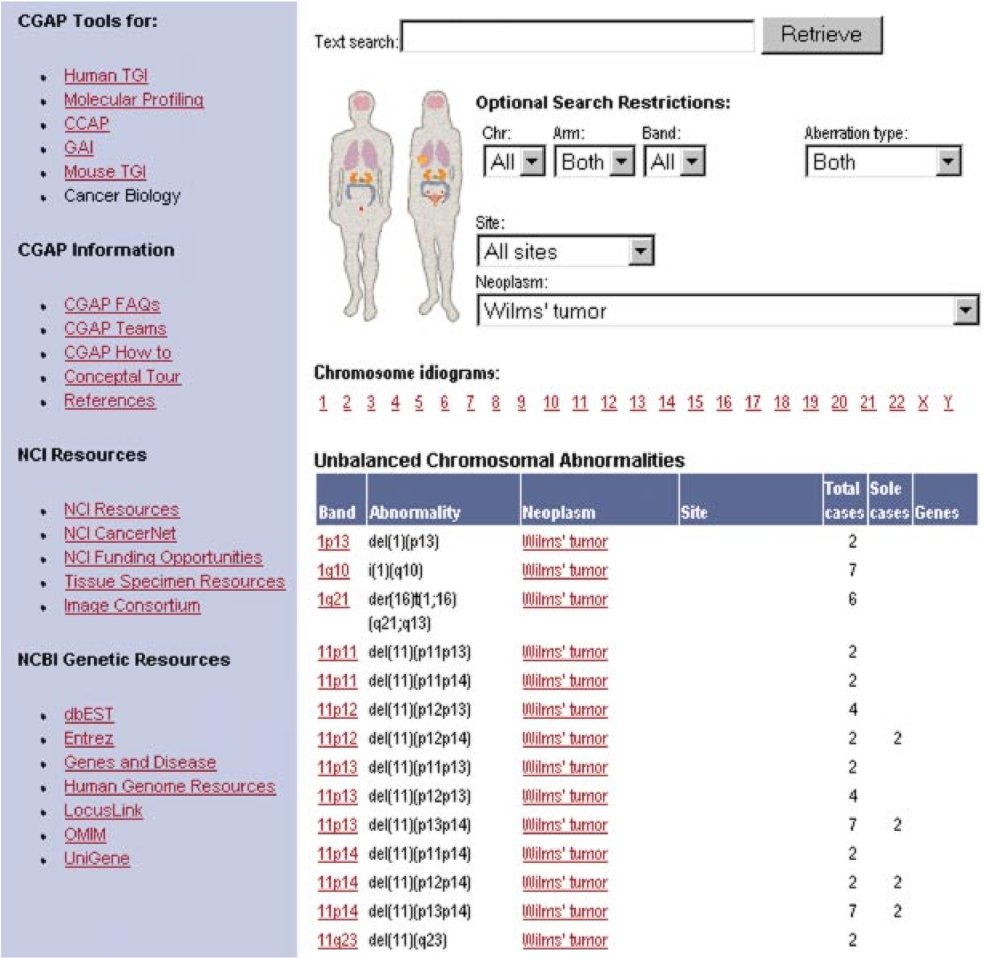

**Figure 2.** The Cancer Chromosome Aberration Project. The Recurrent Chromosome Aberrations page was searched by 'neoplasm' type for 'Wilms' tumour'. The table shows some of the recurrent aberrations identified in cases of Wilms' tumour, organized by chromosome band affected. Note the predominance of deletions involving the 11p11–p14 region corresponding to loss of the WT1 and/or WT2 tumour suppresser loci. Reproduced with the kind permission of the National Library of Medicine **http://www.ncbi.nlm.nih.gov**

quantitative data about expression patterns of mRNA samples from the mass sequencing of 9–10 base tags corresponding to 3′ regions of transcripts (see **www.ncbi.nlm.nih.gov/SAGE** and **http://www.SAGEnet.org** for detailed descriptions of the technique and its drawbacks). The technique has advantages over microarray approaches as it surveys unknown transcripts without the need to isolate a physical clone, although the allocation of tags to genes can be imprecise in some cases. A considerable depth of coverage of tens of thousands of tags can readily be generated, a considerable improvement over the EST sequencing approaches of CGAP for comparative analysis of expression levels of genes in different samples. A large number of SAGE analyses have been performed on >80 different cancer cell lines, with an average coverage of 50 000 tags per library.

Two example differential analyses, of colon and brain tumour against normal tissue, are presented at **www.ncbi.nlm.nih.gov/SAGE**. Following these links takes you to the comparison and analysis page for these experiments (Figure 3), where the relative abundance levels of the indicated tags and confidence limits are given. Links to the SAGE libraries provide details of library preparation, numbers of tags and corresponding genes, etc. The

Copyright © 2001 John Wiley & Sons, Ltd. 

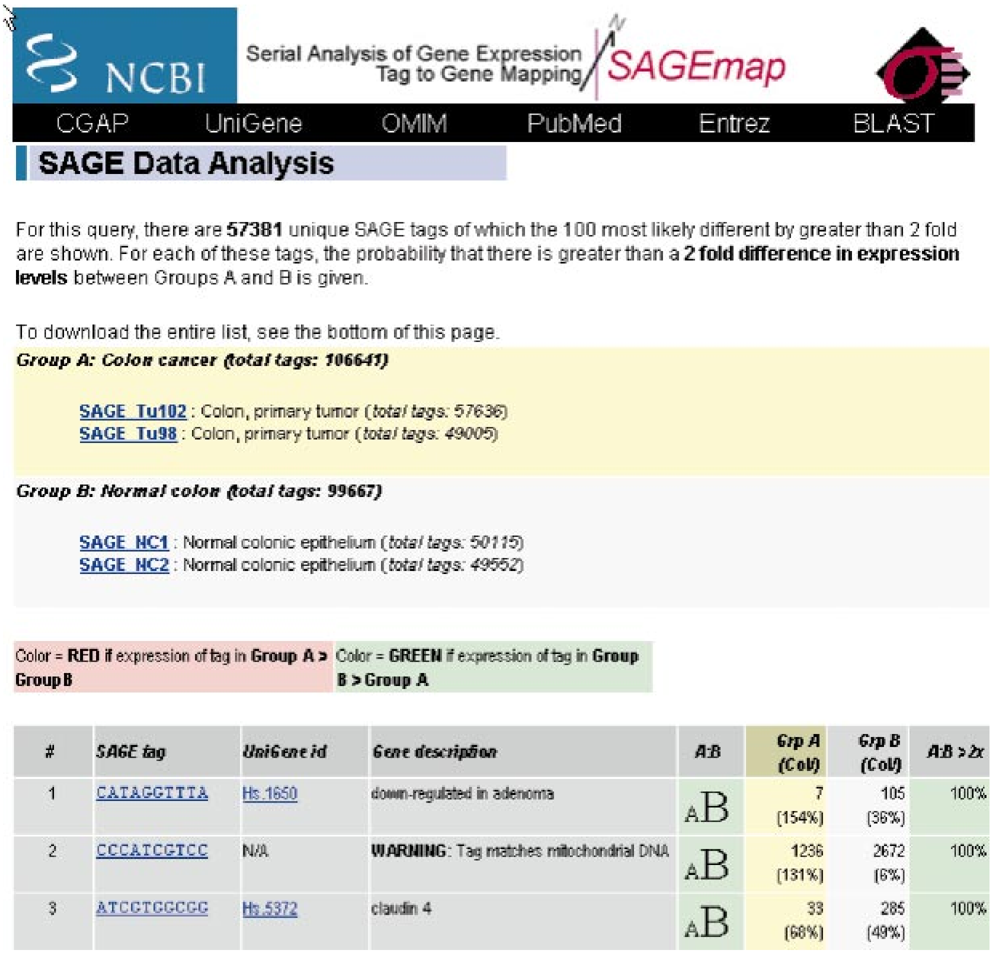

NCBI

Serial Analysis of Gene Expression Tag to Gene Mapping SAGEmap

CGAP UniGene OMIM PubMed Entrez BLAST

SAGE Data Analysis

For this query, there are **57381** unique SAGE tags of which the 100 most likely different by greater than 2 fold are shown. For each of these tags, the probability that there is greater than a **2 fold difference in expression levels** between Groups A and B is given.

To download the entire list, see the bottom of this page.

***Group A: Colon cancer (total tags: 106641)***

SAGE_Tu102 : Colon, primary tumor (*total tags: 57636*)
SAGE_Tu98 : Colon, primary tumor (*total tags: 49005*)

***Group B: Normal colon (total tags: 99667)***

SAGE_NC1 : Normal colonic epithelium (*total tags: 50115*)

SAGE_NC2 : Normal colonic epithelium (*total tags: 49552*)

Color = **RED** if expression of tag in **Group A > Group B**

Color = **GREEN** if expression of tag in **Group B > Group A**

| # | *SAGE tag* | *UniGene id* | *Gene description* | *A:B* | *Grp A (Col)* | *Grp B (Col)* | *A:B > 2x* |
|---|---|---|---|---|---|---|---|
| 1 | CATAGGTTTA | Hs.1650 | down-regulated in adenoma | AB | 7 [154%] | 105 [36%] | 100% |
| 2 | CCCATCGTCC | N/A | **WARNING**: Tag matches mitochondrial DNA | AB | 1236 [131%] | 2672 [6%] | 100% |
| 3 | ATCGTGGCGG | Hs.5372 | claudin 4 | AB | 33 [68%] | 285 [49%] | 100% |

**Figure 3.** Serial Analysis of Gene Expression (SAGE) colon cancer vs. normal colon comparison. The constituent SAGE libraries in each of the comparator groups A and B are shown, with links to associated library information. The first three tags (of many) are shown in the results table. Clicking on the SAGE tag reveals the abundance of that tag in all the different SAGE libraries available via the NCBI. The first tag, corresponding to UniGene Hs. 1650 'downregulated in adenoma' DRA gene, was sequenced seven times in the group A libraries and 105 times in the group B libraries. The A : B column shows that all of these three tags are more abundant in the normal colon epithelium libraries (group B) and allows rapid identification of tags that are up- or downregulated in the different libraries. Reproduced with the kind permission of the National Library of Medicine **http://www.ncbi.nlm.nih.gov**

levels of expression of a given gene in the SAGE libraries can be analysed from a 'Virtual Northern' button, which identifies potential SAGE tags from a given sequence, which must include the 3′ terminal sequence of the transcript. These tags can then be followed to show abundance levels in different SAGE libraries, UniGene clusters and links to the SAGE libraries themselves. While all possible tags are indicated, in general only one of these is likely to be generated from the SAGE technique (generally the tag nearest the 3′ terminal end of the gene). Any of the SAGE libraries available at NCBI can be compared using the SAGEmap Xprofiler tool individually, or as pooled groups—the two colon tumour samples and two normal colon samples in Figure 3 have been analysed in this manner, but more than two samples can be compared.

### Microarray analysis

Microarray analysis does not suffer from the limitations of depth of sample coverage that

Copyright © 2001 John Wiley & Sons, Ltd. 

restricts cDNA library sequencing and to a lesser extent SAGE, and can be considered the technique that is the most likely to determine representative expression levels of given genes. However, the limitation of microarray analysis is that it is only possible to detect a sequence that is included on the array, and whole human genome arrays are not yet practicable, although they may become so in the next few years with the completion of the Human Genome Project and the increased miniaturization capability of array technology.

The laboratory of Patrick Brown at Stanford University is one of the leaders in the field of cDNA microarray analysis of cancer. The Stanford Microarray Database (**http://genome-www4.stanford.edu/MicroArray/SMD/**) has links to some of the landmark papers in the field of microarray analysis of cancer (Alizadeh *et al.*, 2000; Ross *et al.*, 2000), including the large-scale NCI analysis of 60 cancer cell lines and drug sensitivities (Scherf *et al.*, 2000; see also **http://discover.nci.nih.gov/nature2000/**). These huge datasets can be browsed or searched to track expression changes in genes represented on their microarrays across up to 60 common cancer cell lines. The individual databases for each of their key publications can also be browsed or searched individually, using the GeneXplorer web server to track expression levels of individual genes across the complete datasets. The site also has links to other microarray sites, and to the Stanford Genomics Resource for genomics information for a variety of model organisms.

### Proteomics

The application of proteomics technology to the study of cancer is another burgeoning area of interest. Several groups have begun to generate and collate proteomics databases on the web (e.g. Ludwig Institute **http://www.ludwig.ucl.ac.uk/st/elec.html,** and the NCI protein expression database (**http://www.nci.nih.gov/intra/lmp/jnw/Prot.html;** Myers *et al.*, 1997) and there are indexes of these resources (available at **http://expasy.cbr.nrc.ca/ch2d/2d-index.html** and **http://www-lecb.ncifcrf.gov/ips-databases.html**).

I shall focus on one resource in particular, the bladder cancer database from the laboratory of Julio Celis at the Danish Centre for Human Genome Research in Aarhus (**http://biobase.dk/cgi-bin/celis**). This site includes an excellent introduction to bladder cancer pathology and aetiology and describes their research interests and experimental considerations of proteomics in cancer research. The site also includes movie and text protocols for sample preparation and running 2D gels. The other principal interest of this group is keratinocyte biology, which is in fact currently covered in more detail than bladder cancer.

The bulk of the site is dedicated to the *2D PAGE Databases and Galleries* (Celis *et al.*, 1998). The *Human 2D PAGE Databases* link summarizes the gel data obtained from different keratinocyte or bladder cancer samples, with links to a representative gel image for each cell or sample type (Figure 4). Spots that have been identified are marked with red crosses, and are linked to summary information for that protein, which includes a brief description of the protein with size and pI values, a list of the tissues/samples expressing this protein, method of identification and links to other database entries, including SWISS-PROT, UniGene and OMIM, if available. Spots that are not marked have not yet been identified and clicking on them links to a spot identification number, the approximate size and pI, and expression information. Gel databases can also be interrogated to search for your favourite protein or keyword, or browsed by protein name. There is also a *Mouse 2D PAGE Database*, which is currently more limited but includes links to mouse genome and transgenic databases. There are multiple normal mouse tissue samples in the *ZOO 2D Gel Gallery*.

The *Human 2D Gel Gallery* links for human and mouse lead to a wide range of tissue/cell or organelle sample images and some side-by-side comparisons. These comparisons have some differences indicated (Figure 5), which are linked to the protein description database, although spots that are not marked cannot be followed from this comparison view.

A wide range of antibodies have been used to probe different cell line samples, and these images can be viewed from the *2D Gel Immunoblots* link, sorted by antibody description. The corresponding protein spots indicated are also linked to the protein database.

### Other links to cancer resources on the web

There are a number of sites that have collated indexes of links to more cancer sites (such as

Copyright © 2001 John Wiley & Sons, Ltd. 

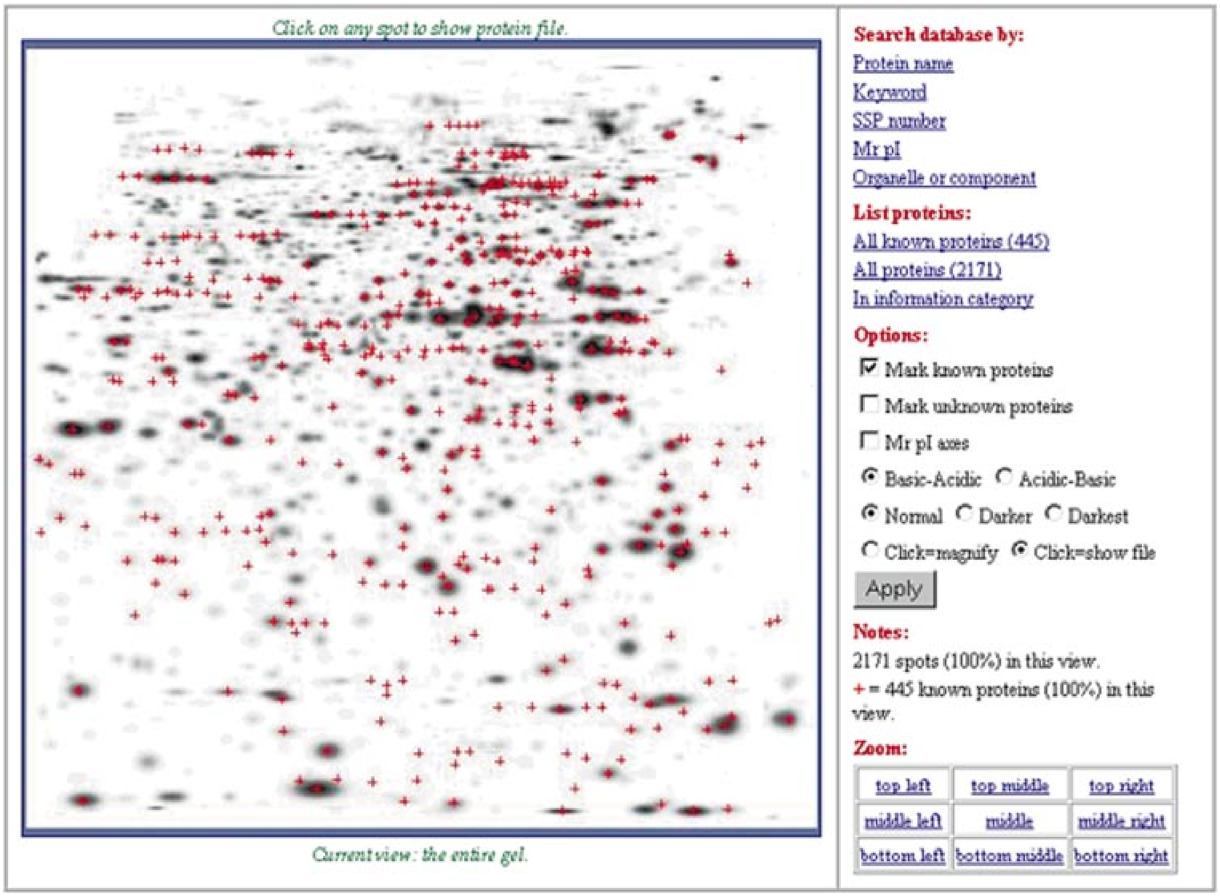

**Figure 4.** The Danish Centre for Genome Research Transitional Cell Carcinoma (TCC) 2D PAGE database. This image shows the master template for a TCC 2D gel. In this view, all spots that have been identified and correspond to known proteins are marked with a red cross, while unmarked spots correspond to unknown proteins. The database can be searched or browsed by following the links in the top right of this image. Reproduced with kind permission of Julio E. Celis of The Danish Centre for Genome Research **http://biobase.dk/cgi-bin/celis**

**www.cancerindex.org/clinks19.htm#special**). Many of the sites I have discussed in this review also include links to additional sites in their fields of interest. Of particular interest is **http://tumor.informatics.jax.org/cancer_links.html**—a comprehensive set of links to cancer resources on the web from the Jackson Laboratory, Maine, USA (see also Bult *et al.*, 1999). Links are grouped by: animal models (descriptions of models and phenotypes as well as sources for animals); cancer genetics and genomics; cancer biology; pathology; reagents; services; and protocols. An index of the research resources available from the NCI, including animals, specimens, cell lines, etc., is given at **http://resresources.nci.nih.gov**.

### Discussion

It is clear that postgenomic biology has a tremendous amount to offer to cancer research, and that there are a host of resources available via the Internet to access this information. The technology is improving all the time and it is now becoming possible to obtain a level of description of a cancer cell undreamed of a few years ago. Some of the latest techniques for profiling cancer cells at the transcriptional, proteomic and molecular pathway levels are reviewed in Liotta and Petricoin (2000) and indicate the exciting potential for making tremendous advances in cancer research that will undoubtedly be fulfilled in the years to come.

Copyright © 2001 John Wiley & Sons, Ltd. 

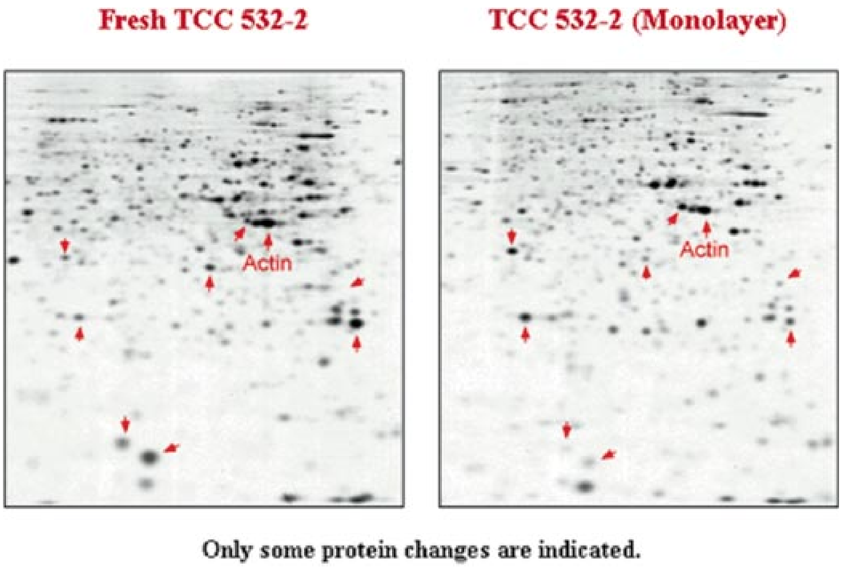

**Figure 5.** An example comparison of 2D profiles from the Human 2D Gel Gallery database at the Danish Centre for Genome Research. Samples on the gels are derived from a bladder cancer transitional cell carcinoma (TCC). One sample was generated from cells freshly derived from a patient, while the second sample was generated from a monolayer culture of a primary cell line derived from the same patient sample. A number of spots showing differential abundance between the two samples are indicated by small red arrows. These arrows are links to the protein files from one of the 2D PAGE Gel Databases (in this case the keratinocyte database). Reproduced with the kind permission of Julio E. Celis of The Danish Centre for Genome Research

I hope that this review has given a taste of some of the resources available on the Internet that can be used to facilitate cancer research in the post-genomic era. There is a lot more information out there, so start looking!

### References

Alizadeh AA, Eisen MB, Davis RE, *et al*. 2000. Distinct types of diffuse large B-cell lymphoma identified by gene expression profiling. *Nature* **403**: 503–511.

Bult CJ, Krupke DM, Tennent BJ, Eppig JT. 1999. A survey of web resources for basic cancer genetics research. *Genome Research* **9**: 397–408.

Bult CJ, Krupke DM, Sundberg JP, Eppig JT. 2000. Mouse Tumor Biology Database (MTB): enhancements and current status. *Nucleic Acids Res* **28**: 112–114.

Celis JE, Østergaard M, Jensen NA, Gromova I, Rasmussen HH, Gromov P. 1998. Human and mouse proteomic databases: novel resources in the protein universe. *FEBS Lett* **430**: 64–72.

Liotta L, Petricoin E. 2000. Molecular profiling of human cancer. *Nature Genet Rev* **1**: 48–56.

Myers TG, Anderson NL, Waltham M, *et al*. 1997. A protein expression database for the molecular pharmacology of cancer. *Electrophoresis* **18**: 647–653.

Ross DT, Scherf U, Eisen MB, *et al*. 2000. Systematic variation in gene expression patterns in human cancer cell lines. *Nature Genet* **24**: 227–235.

Scherf U, Ross DT, Waltham M, *et al*. 2000. A gene expression database for the molecular pharmacology of cancer. *Nature Genet* **24**: 236–244.

Velculescu VE, Zhang L, Vogelstein B, Kinzler KW. 1995. Serial analysis of gene expression. *Science* **270**: 484–487.

Wixon JL. 2000. The Jackson Laboratory Mouse Genome Informatics site: version 2.3.2. *Yeast* **17**: 134–145.

---

Some of the sites reviewed will already be known to you but perhaps their content will be less well-known. The Website Review is intended to help you discover new sites of interest, but also to provide a rapid and convenient means of revealing what you always knew was there but never had the time or inclination to look at. These articles are a personal critical analysis of the website. If you have any information about sites you think are worthy of being more widely known, the Managing Editor would be pleased to hear from you.

Copyright © 2001 John Wiley & Sons, Ltd.
